# Hypophosphatemic vitamin D-resistant osteomalacia: A case report

**DOI:** 10.3892/etm.2013.1209

**Published:** 2013-07-08

**Authors:** MIN WANG, XIA CAO, BINGZHEN CAO

**Affiliations:** Department of Neurology, Jinan Military General Hospital, Jinan, Shandong 250031, P.R. China

**Keywords:** adult-onset hypophosphatemic vitamin D-resistant osteomalacia, tumor-induced osteomalacia, thyroid, parathyroid

## Abstract

This study concerns the case of a 59-year-old woman who was admitted to hospital after experiencing systemic bone pain and muscle weakness for more than 2 years. The patient was diagnosed with numerous fractures via bone imaging emission computed tomography (ECT) and hypophosphatemia. Laboratory results showed a high alkaline phosphatase concentration. Thyroid nodules were visible on the thyroid and parathyroid glands during ultrasound examinations. However, no symptomatic or biochemical improvement was observed following a thyroid nodulectomy. The patient was administered large doses of neutral phosphate preparations, vitamin D3 and calcium. A gradual improvement in the condition of the patient was observed. For bone pain associated with multiple fractures in elderly patients, calcium and phosphorus metabolism disorders, and active vitamin D deficiency should be considered and an early diagnosis should be performed.

## Introduction

Adult-onset hypophosphatemic vitamin D-resistant osteomalacia (AHVDRO) is a group of diseases characterized mainly by poor bone mineralization, osteomalacia or rickets (caused by hypophosphatemia) and insufficient active vitamin D production. There are three forms of AHVDRO: X-linked hypophosphatemic rickets/osteomalacia (XLH), autosomal dominant hypophosphatemic rickets (ADHR) and tumor-induced osteomalacia (TIO). Previous studies have investigated other factors that may contribute to the development of hypophosphatemia-associated osteomalacia, such as vitamin D receptor resistance. In the current study we present a patient with bone pain associated with multiple fractures. Large doses of neutral phosphate preparations, vitamin D3 and calcium improved the patient's symptoms. For bone pain associated with multiple fractures in elderly patients, calcium and phosphorus metabolism disorders and active vitamin D deficiency should be considered and an early diagnosis should be established. The presetn study was approved by the Ethics Committee of Jinan Military General Hospital, Jinan, China.

## Case report

A 59-year-old female was admitted to Jinan Military General Hospital (Jinan, China) on March 3, 2010 after experiencing sternocostal, back and bilateral groin pain, and progressive lower-extremity weakness for more than 2 years. The cause of the pain was not evident initially. The patient described the pain as being intermittent and dull. Gradually, both lower extremities became weaker, weakness and pain were aggravated following exercise and labor and relieved by rest, and the condition worsened. However, the patient did not present morning stiffness, low fever or night sweats, and the extent of pain was not affected by weather changes. At 1 year prior to admission, magnetic resonance imaging (MRI) of the lumbar vertebrae had been performed on the patient, which revealed L3/4 and L4/5 disk herniation. MRI of the thoracic vertebra showed ligamentum flavum hypertrophy and calcification at the T6/7 vertebral level, resulting in spinal cord compression. A conservative treatment regimen was initiated, but there was no improvement in the severity of the symptoms. The patient had increasing difficulty rolling over and getting up, and upon hospitalization, the patient was not able to walk independently. Thus, the patient was admitted to the Department of Osteopathy, Jinan Military General Hospital for thoracic spinal stenosis.

**Figure 1:**
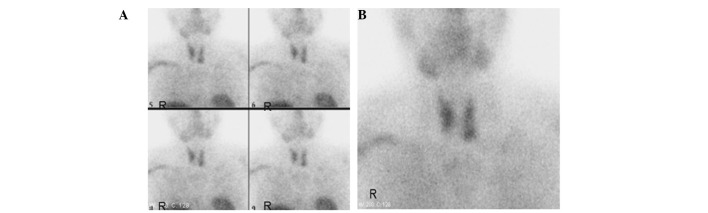
Parathyroid emission computed tomography. (A) Blood pool images and (B) a 15 min static image of the neck.

Osteopathic surgeons considered that the cause of the clinical manifestations was unlikely to be thoracic spinal stenosis and tests on anti-neuron antibodies [anti-Hu, 70.080 ng/ml (normal, <76.8 ng/ml); anti-Ri, 31.7412 ng/ml (normal, <26.5 ng/ml) and anti-Yo, 16.598 ng/ml (normal, <18.0 ng/ml)] showed that the concentrations of anti-Hu and anti-Ri were significantly increased above normal levels. Therefore, the patient was diagnosed with suspected paraneoplastic syndrome and was transferred to the Department of Neurology, Jinan Military General Hospital. Following admission, heart, lung and abdominal examinations showed no significant abnormities. Neurological examinations showed that the patient had full consciousness and fluent speech, cranial nerve examination was normal, double upper-limb myodynamia classified as Grade V, double lower-limb distal myodynamia classified as Grade III- and proximal myodynamia classified at Grade V-. The muscle tone of all four limbs was normal, the double upper-limb tendon reflex was symmetrical and normal, the double lower-limb tendon reflex was symmetrical and reduced, and the finger-to-nose test was performed stably and accurately. However, the heel-knee-tibia test on the two lower limbs could not be completed. Sensation examination showed no significant abnormities, the test for pathological reflexes was negative, the neck was soft and flexible, and meningeal irritation signs were negative. The pressing pain at the sternum, multiple ribs, bilateral hip joints and groin was significant. Since the onset of the illness, drinking, eating, urination and defecation were normal, and the body weight had not reduced significantly. Past medical history and family history revealed nothing of note. The results of blood examinations were normal (red blood cells, 3.31×10^12^/l; hemoglobin, 95 g/l; and platelets, 568×10^9^/l). The alkaline phosphatase level was elevated [339 U/l (normal, 26–150 U/l)], the blood phosphorus level was reduced [0.64 mmol/l (normal, 0.80–1.60 mmol/l)] and the calcium [2.22 mmol/l (normal, 2.10–2.60 mmol/l)] and magnesium concentrations were normal. The levels of tumor-marker antibodies [sialic acid: 82.1 mg/dl (normal, 45.6–75.4 mg/dl), carcinoembryonic antigen (CEA), α-fetoprotein (AFP), carbohydrate antigen 72-4 (CA72-4), carbohydrate antigen 12-5 (CA12-5), carbohydrate antigen 19-9 (CA19-9), carbohydrate antigen 15-3 (CA15-3), cytokeratin 19 fragment (CY21-1) and neuron-specific enolase (NSE)] were within the normal range, as were the erythrocyte sedimentation rate (31 mm/h), serum protein electrophoresis, blood parathormone level [21.55 ng/l (normal, 15–65 ng/l)] and albumin levels. Results from the 5-item thyroid function test [thyroid stimulating hormone, (TSH); free triiodothyronine (FT3); free thyroxine (FT4); thyroid peroxidase antibodies (TPO); and thyroglobulin antibody (TGAb)], 6-item sex hormone test (estradiol, progesterone, testosterone, prolactin, luteinizing hormone and follicle stimulating hormone), 5-item hepatitis B and C virus tests and HIV antibody test did not show any anomalies, and the Bence Jones urinary protein test was negative. Bone marrow puncture, gynecological ultrasonography, abdominal ultrasonography, echocardiography and double hip-joint computed tomography (CT) scan showed no obvious abnormalities. Electromyography showed no characteristic changes and the repetition-frequency stimulation test showed no significant alternation. Pulmonary CT demonstrated an increase in double lung markings, the presence of pleural thickening and calcification in the right liver lobe. Thyroid color ultrasonography revealed multiple thyroid nodules, localized neoplasia, locally coexisting Hashimoto's disease, the nature of left nodule was undetermined, parathyroid lesions could not be excluded and cervical lymph nodes were swollen. No significant abnormal nuclide intake was demonstrated following bilateral parathyroid ECT examination (Fig. 1) and clinical tests repeatedly showed that the parathormone and 24-h urine phosphorus and urine calcium levels were normal. Therefore, a diagnosis of a parathyroid disease was not considered.

**Figure 2:**
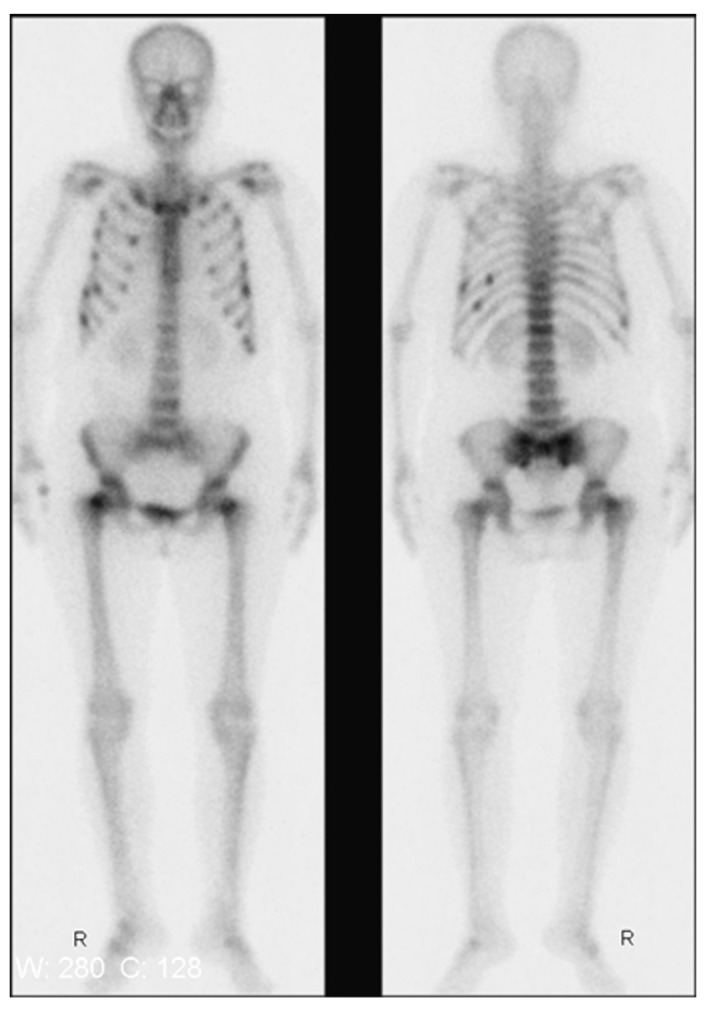
Whole-body bone imaging emission computed tomography. Whole-body positron-emission tomography CT (PET-CT) examination showed that several ribs and double femoral neck (incompletely) had discontinuity and numerous old fracture lines (loose lines) were present, in addition to mild T12 compression changes. Bilateral hip joint swelling was accompanied by an increase in FDG metabolism and these regions were classified as inflammatory hip lesions.

**Figure 3:**
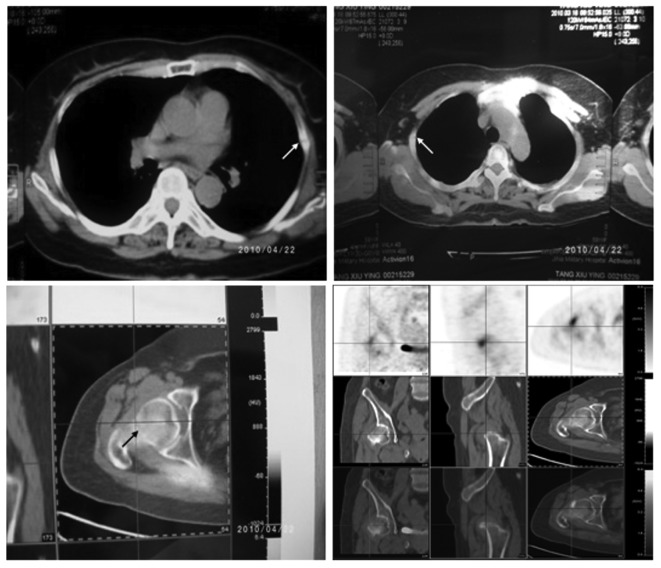
Whole body PET-CT examination. Several ribs and double femoral neck (incompletely) had continuous discontinuity and numerous old fracture lines were present (indicated by arrows indicate loose lines). PET-CT, positron-emission tomography-computed tomography.

Whole-body bone emission computed tomography (ECT; via the intravenous administration of ^99^ mTc - MDP) showed that nuclide intake was increased in several locations (Fig. 2), and the cause was considered to be malignant. Whole-body positron-emission tomography CT (PET-CT) examination (Fig. 3) showed that several ribs and double femoral neck (incompletely) had discontinuity and numerous old fracture lines (loose lines) were present, as well as mild T12 compression changes. Local fluorodeoxyglucose (FDG) metabolism increased in the cervical vertebra-2 accessory bone, but did not increase in the regions of reduced local density of the left thyroid gland, which were considered to be thyroid nodules. Bilateral hip joint swelling was accompanied by an increase in FDG metabolism and these regions were classified as inflammatory hip lesions. Bone mineral density tests showed that left forearm bone density had reduced by 17.96% [T-score: −1.43 (−1>T-score >−2.5 is suggestive of bone loss)]. The results of blood phosphorus tests (0.48–0.64 mmol/l) led to the clinical consideration that the patient may be diagnosed with hypophosphatemic osteomalacia. However, re-examination of the patient following the administration of sodium glycerophosphate and Rocaltrol^®^ (Roche, Shanghai, China) demonstrated that the treatment was ineffective (the blood phosphorus concentration did not increase). Tests of blood from the right subclavian vein indicated that the concentration of fibroblast growth factor (FGF)-23 was 32.44 ng/l (normal, 40–90 ng/l).

**Figure 4:**
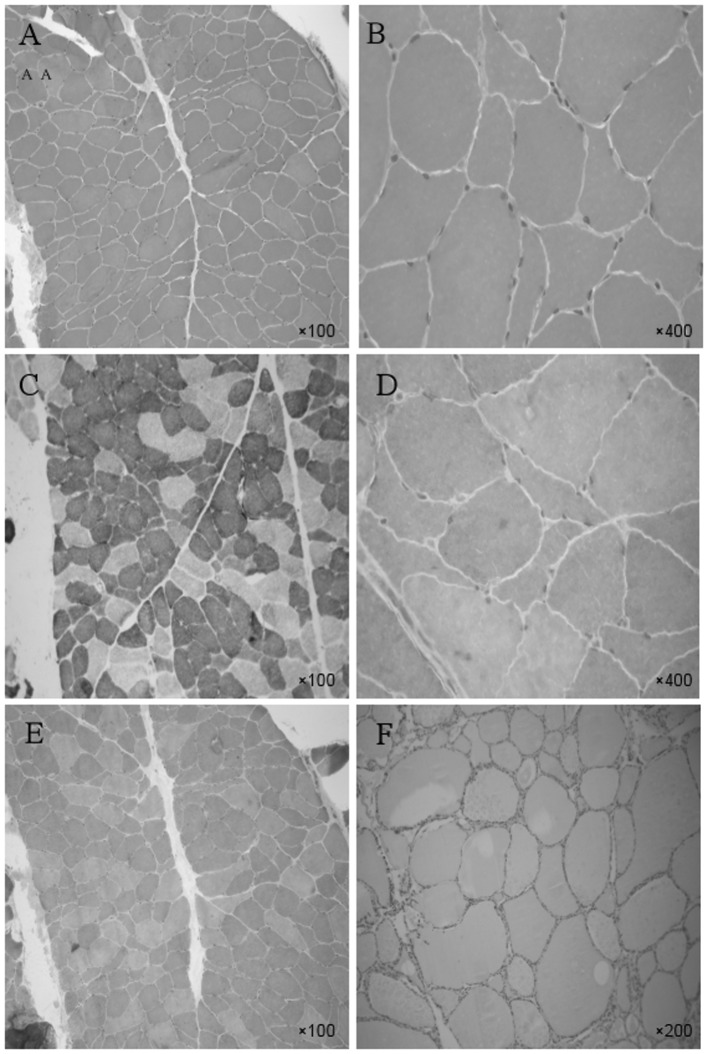
Tibialis anterior muscle biopsy. H&E staining: Single or small angular atrophic muscle fibers were apparent but no necrotic muscle fibers or inflammatory cellular infiltrations were observed at (A) magnification, ×100 or (B) magnification, ×400. (C) Nicotinamide adenine dinucleotide tetrazolium oxidoreductase (NADH-TR) staining: The proportions of the two types of muscle fibers were approximately normal, although they were moderately distributed in groups according to muscle fiber type, and the atrophic muscle mainly comprised type I fibers (magnification, ×100). MGT staining (D) magnification, ×400 and (E) magnification, ×100, and (F) Oil Red O staining, magnification, ×200, showed no abnormalities. H&E, hematoxyin and eosin.

**Figure 5:**
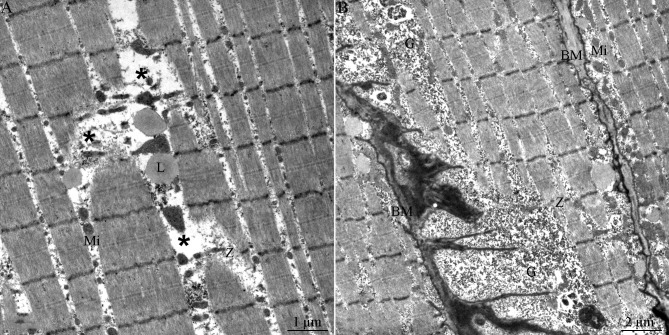
Tibialis anterior muscle biopsy electron microscopy. Electron microscopy results showed fractured or missing muscle fibers (*) and the presence of lipid grains (L) between the fractured muscle fibers. The muscle fibers had a sparse transverse arrangement and relatively few mitochondria (Mi). Sarcomere (Z marked) is the same length, vertical arrangement is still regular. Muscle satellite cells can be seen in basement membrane(BM). In addition, an accumulation of glycogenosomes (G) was observed beneath the muscle cell membrane.

To ensure a definitive diagnosis, a tibialis anterior muscle biopsy was conducted on March 27, 2010 (Fig. 4). Single or small angular atrophic muscle fibers were apparent but no necrotic muscle fibers or inflammatory cell infiltration were observed (Fig. 4A and B). The proportion of the two types of muscle fibers was approximately normal, although there was some clustering of the muscle fiber types and the atrophic muscle mainly comprises type I fibers (Fig. 4C). Gomori staining (Fig. 4D and E) and Oil Red O staining (Fig. 4F) showed no abnormalities. Electron microscopy results (Fig. 5) showed fractured or missing muscle fibers and the presence of lipid grains between the fractured muscle fibers. The muscle fibers had a sparse transverse arrangement and relatively few mitochondria. In addition, an accumulation of glycogenosomes was observed beneath the muscle cell membrane.

**Figure 6:**
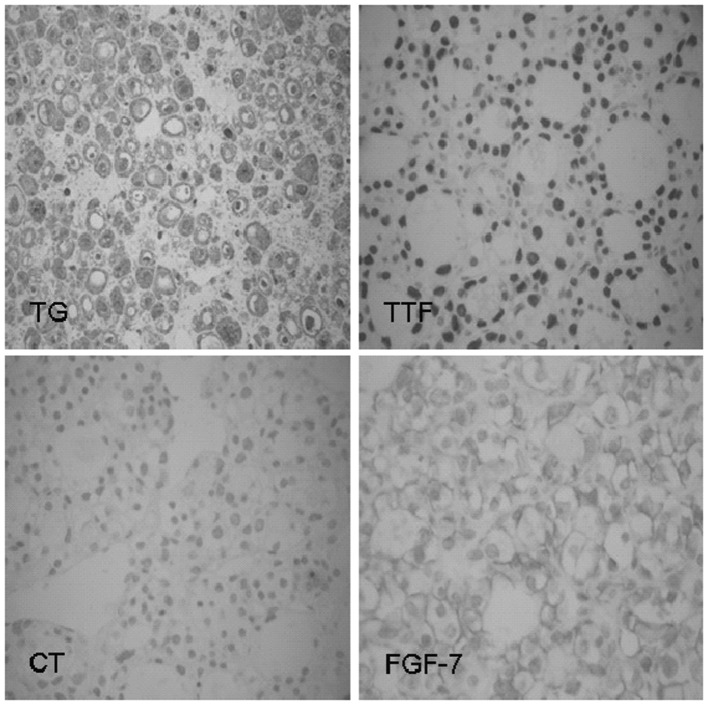
Pathological results of thyroid nodules. Partial follicular lesions had adenomatous hyperplasia, with rich cells and active growth. The results of immunohistochemical staining were positive for thyroid transcription factor (TTF)-1, fibroblast growth factor (FGF)-7 and thyroglobulin (TG) and negative for calcitonin (CT).

On April 16, 2010, a thyroid nodulectomy was performed to exclude TIO as the cause of the symptoms. The postoperative pathological results showed nodular goiter in the left lobe. Partial follicular lesions had adenomatous hyperplasia, with abundant cells and active growth. The results of immunohistochemical staining (Fig. 6) were positive for thyroid transcription factor-1 (TTF-1), CK8/18, FGF-7 and thyroglobulin (TG), and negative for calcitonin (CT). At 1 week after the surgery, although the sternal pain had been alleviated, the patient continued to experience significant pain in the bilateral rib arch, back and groin, and was unable to roll over, get up or walk. A re-examination was performed on May 20, 2010 and the whole-body bone imaging ECT results showed no significant changes. The results of a postoperative re-examination were as follows; anti-Hu, anti-Ri and anti-Yo antibody levels were normal, the erythrocyte sedimentation rate was 25 mm/h, post-operative continuous phosphorus supplementation was ineffective (phosphorus: 0.48–0.56 mmol/l), calcium and magnesium levels were normal, and the 24-h urine calcium and urine phosphorus concentrations were normal. Following thyroid tumor resection, the condition of the patient did not improve significantly, therefore, a diagnosis of TIO was not considered. The vitamin 1,25-(OH)_2_D3 concentration was significantly lower than normal [<4.00 ng/ml (normal concentration: ≥30.00 ng/ml)], which was considered to be caused by hypophosphatemic vitamin D-resistant osteomalacia which was subsequently treated with neutral phosphate preparations (73.1 g disodium hydrogen phosphate and 6.4 g potassium dihydrogen phosphate in an aqueous solution of final volume 1,000 ml). Every 4 h, 20 ml of the preparation was administered orally (5 times daily), in addition to a 1-ml vitamin D3 intramuscular injection once every 2 weeks (or oral vitamin D3 tablets and calcium). The condition of the patient improved gradually, and after 2 months, the sternocostal, back and groin pain was relieved significantly. The phosphorus concentration at the 6-month follow-up visit was 0.79 mmol/l, and had increased to 0.98 mmol/l at the 1-year follow-up. The whole-body pain had ceased and the patient was able to roll over, get up alone and walk slowly with assistance. When walking, the patient continued to experience weakness in both legs, but to a lesser extent than previously.

## Discussion

The patient had no relevant family history to aid in diagnosis confirmation and therefore, it was considered a sporadic case. ADHR and XLH were excluded as possible causes, and a diagnosis of TIO was considered initially. TIO is a rare paraneoplastic syndrome, an acquired form of hypophosphatemic osteomalacia caused by the excessive secretion of phosphorus-regulating factors and an increase in renal phosphorus excretion. In 1980, China reported the first case of TIO caused by groin mesenchymoma (1). To date, fewer than 200 cases have been reported worldwide. TIO has several typical clinical manifestations. First, bone pain develops progressively, mainly in the arms and legs and at weight-bearing joints. Second, the excretion of phosphorus in the urine increases significantly and blood phosphorus levels are significantly reduced, while blood calcium levels remain normal. Third, the administration of conventional phosphorus and vitamin D supplements has almost no effect; high-dose phosphorus and vitamin D supplementation is usually required. Fourth, alkaline phosphatase levels increase in the blood. Fifth, vitamin 1,25-(OH)_2_D3 levels decrease; in certain cases, this is accompanied by secondary hyperparathyroidism. Finally, a relevant tumor is usually present and the determinant of TIO, although it is often hidden and of small size. Therefore, following tumor resection, symptoms such as blood phosphorus levels may rapidly and significantly improve. Radiographs of patients with TIO typically show reduced bone densities, as well as vague deformations in the bone trabecula, pelvis and vertebra, and bone fractures or pseudo-fracture formations are present in numerous cases (2). In the current case, following thyroid nodulectomy, no significant improvement of the clinical symptoms was observed and blood phosphorus levels did not increase significantly. Therefore, a diagnosis of TIO was not made.

Studies have shown that FGF-23 is associated with the onset of hypophosphatemic osteomalacia; it is a known phosphorus-regulating factor (3,4), with a normal level of ~10–50 ng/l. The tumor in TIO patients may express and secrete large quantities of FGF-23 (5), which reduces renal phosphate reabsorption and increases the amount of phosphorus excreted in the urine (6). In addition, an increase in FGF-23 concentrations may inhibit the production and activity of 1-α hydroxylase, thereby reducing the production of 1,25-(OH)_2_D3 and phosphorus. Following tumor resection, FGF-23 levels may quickly decrease (7). As test results showed that FGF-23 concentrations were normal in this patient, a diagnosis of TIO was not supported. Therefore, since calcium levels were normal, phosphorus levels were reduced, alkaline phosphatase levels were increased, urine phosphorus concentrations were normal or increased, and there was no medical history of deficiency in vitamin D, azotemia or other renal tubular function insufficiency, a diagnosis of hypophosphatemic vitamin D-resistant osteomalacia (AHVDRO) was considered. The treatment of this disease should initially target the cause and if a tumor is discovered to be the cause, resection may reduce the severity of the disease and biochemical indicators may be completely restored to normal levels (8). If it is not possible to discover or eliminate the cause, symptomatic treatment should be adopted, including the administration of high-dose active vitamin D and phosphorus supplements, as well as calcium supplements. Notably, lifelong treatment is required. Phosphorus should be supplemented every 4–6 h since it is rapidly excreted. During treatment, it is important to re-examine calcium and phosphorus levels regularly to avoid vitamin D intoxication. The prognosis is closely associated with the degree of bone density change (9). In the current study, following treatment, the pain of the patient was alleviated. However, considering the long course of the disease and the severe destruction of the bones, the clinical symptoms are unlikely to be eased completely. Therefore, the early detection, diagnosis and treatment of AHVDRO are likely to significantly improve its prognosis.
